# Creep constitutive model of strain energy density considering the whole process of creep deformation

**DOI:** 10.1371/journal.pone.0326599

**Published:** 2025-07-01

**Authors:** Yuemei Li

**Affiliations:** School of Civil Engineering, Liaodong University, Dandong, China; Indian Institute of Technology Roorkee, INDIA

## Abstract

Aiming at the problem that the traditional creep model is difficult to reflect the characteristics of accelerated creep stage of rock, the influence of stress state on accelerated creep is fully considered, and a constitutive model which can describe viscoplastic creep is established based on strain energy density function. In order to comprehensively improve the accurate description of the three stages of rock creep, based on the traditional Nishihara model, an improved creep model is proposed by connecting a nonlinear viscoplastic element in series. The continuous medium strain energy theory is used to analyze the sudden change process of creep macroscopic mechanical behavior, and the critical strain energy density value is defined as the control parameter to predict the occurrence time of accelerated creep. At the same time, the origin software is used to introduce different creep data for fitting. The results show that the creep constitutive model can fit the rock creep test curve well and identify the parameters. It can better simulate the creep deformation characteristics of rock under different stress levels. It can accurately reflect the nonlinear creep characteristics of the whole process of rock creep. It shows that the newly established creep constitutive model has strong applicability and correctness. It provides a new creep constitutive model for future geotechnical engineering.

## 1 Introduction

When sandstone is subjected to long-term load, its stress, strain and failure characteristics change with time. It shows obvious aging characteristics [1–3]. A large number of engineering practices show that the deformation instability and failure state of rock is not when the load is applied or at the end of the project, but to experience a time when the rock keeps constant load. Because of the creep characteristics of sandstone, the stability of rock engineering is often destabilized [4–6]. Therefore, in order to prevent or reduce the creep failure of sandstone under long-term load, people need to conduct in-depth research on sandstone from various aspects such as strength, deformation and failure. With the expansion of the scale and the increase of the number of rock engineering, the environment in which the rock is loaded is getting worse and worse [7]. Sandstone produces creep phenomenon under long-term load. This is not only related to its own physical properties, but also related to the stress level, temperature, moisture and humidity [8]. Water is an inevitable environmental factor in rock engineering, whether it is underground or open-pit rock engineering. It will accelerate the structural safety and stability of sandstone creep. Studies have shown that water content is an important factor affecting the creep properties of sandstone, especially the structure and mechanical parameters of sandstone [9]. At the same time, the creep characteristics of sandstone control the failure process of a project and the catastrophic failure of sandstone under long-term load [10]. As a brittle material, sandstone has natural defects such as holes or microcracks. Thus, the sandstone exhibits nonlinear evolution characteristics in the loading and failure stages under creep conditions. There is still a precursory feature in the acceleration stage before the failure of sandstone, and the abnormal response before these failures can also be used to analyze the creep theoretical model of rock creep failure [11,12].

Many scholars at home and abroad have studied related fields for a long time, promoted the development of rock rheological mechanics, obtained breakthrough and innovative achievements [13], explored valuable research methods, accumulated experience, and provided reasonable evaluation standards and preventive measures for engineering practice [14]. In 1922, Bignham [15] drew on the research results of Werber and boltzmann, and published a book called “ Flow and Plasticity. “ This marks the formal establishment of a new discipline-rheology. After that, with the efforts of many scholars, China ‘s rheological experimental research, theoretical exploration, and practical services have developed rapidly, and gradually formed a complete theoretical research system. The rock rheological model plays an important role in rock rheology, and it is also the focus and hot topic of extensive research in the field of rheology [16–18].

Yu et al. [19] discovered that moisture is a critical factor influencing the stability of underground engineering. Through experiments on red sandstone under different water-soaking conditions, they found that increases in water content and soaking time significantly reduced the strength and elastic modulus of the rock, while also intensifying its creep deformation and failure processes. Similarly, Lin et al. [20] observed that as water content and stress levels increased, the time-dependent characteristics of rock became more pronounced. These increases notably enhanced instantaneous strain, creep strain, and steady-state creep rate but shortened the dilation and failure time of the rock. Furthermore, during the accelerated creep stage, the creep curve of the rock exhibited a “brittle-ductile” transition, and the long-term strength gradually decreased with increasing water content.Li et al. [21] further reported that water immersion significantly altered the strength and failure mode of mudstone. Prolonged soaking led to the deterioration of its creep properties, substantially reducing both creep failure stress and failure time. However, increased confining pressure helped to restrict crack propagation and delayed the weakening process of the rock. Expanding on this, Li et al. [22] found that a higher water saturation coefficient intensified creep deformation, creep rate, and creep duration. As the water saturation coefficient increased, the lateral instantaneous creep and steady-state creep of shale became more pronounced. Based on experimental results, they developed a nonlinear viscoelastic-plastic unloading creep constitutive model (UCCM). Chen et al. [23] investigated the effects of bedding orientation and water content on the creep behavior of shale. They found that higher bedding angles conferred a greater elastic modulus on the shale, whereas higher water content reduced the elastic modulus and increased the creep rate. Using these findings, they established a creep constitutive model for water-containing layered shale based on the Burgers model.

The above research results have well studied the influence of water content on the creep characteristics of rock. The rock creep model under the corresponding conditions is established. However, there is still controversy about how to distinguish and simulate the creep acceleration stage. At present, most of the obtained formulas are empirical formulas, and the theoretical research is not enough. Some of them need a lot of experiments to obtain their constitutive formulas, and the results are difficult to promote. Therefore, based on the strain energy theory, this paper studies the creep fracture process, and proposes a nonlinear creep unified constitutive model based on Perzyna viscoplastic theory combined with Nishihara model.

## 2 A new nonlinear creep model of rock

### 2.1 Creep model under one-dimensional stress state

The Perzyna viscoplastic theory can well simulate the nonlinear viscoplastic mechanical behavior of materials. Its expression can be expressed as [24]


$$\varepsilon_{vp}^{\prime }=\eta_{vp}\;\left\langle \varphi \;\left(F\right)\right\rangle \;\left\{m\right\}$$ (1)

where *η*_*vp*_ is the viscoplastic viscosity coefficient, *φ*(*F*) is an arbitrary function of *F* yield function, {*m*} is the direction of viscoplastic flow.

**Fig 1:**
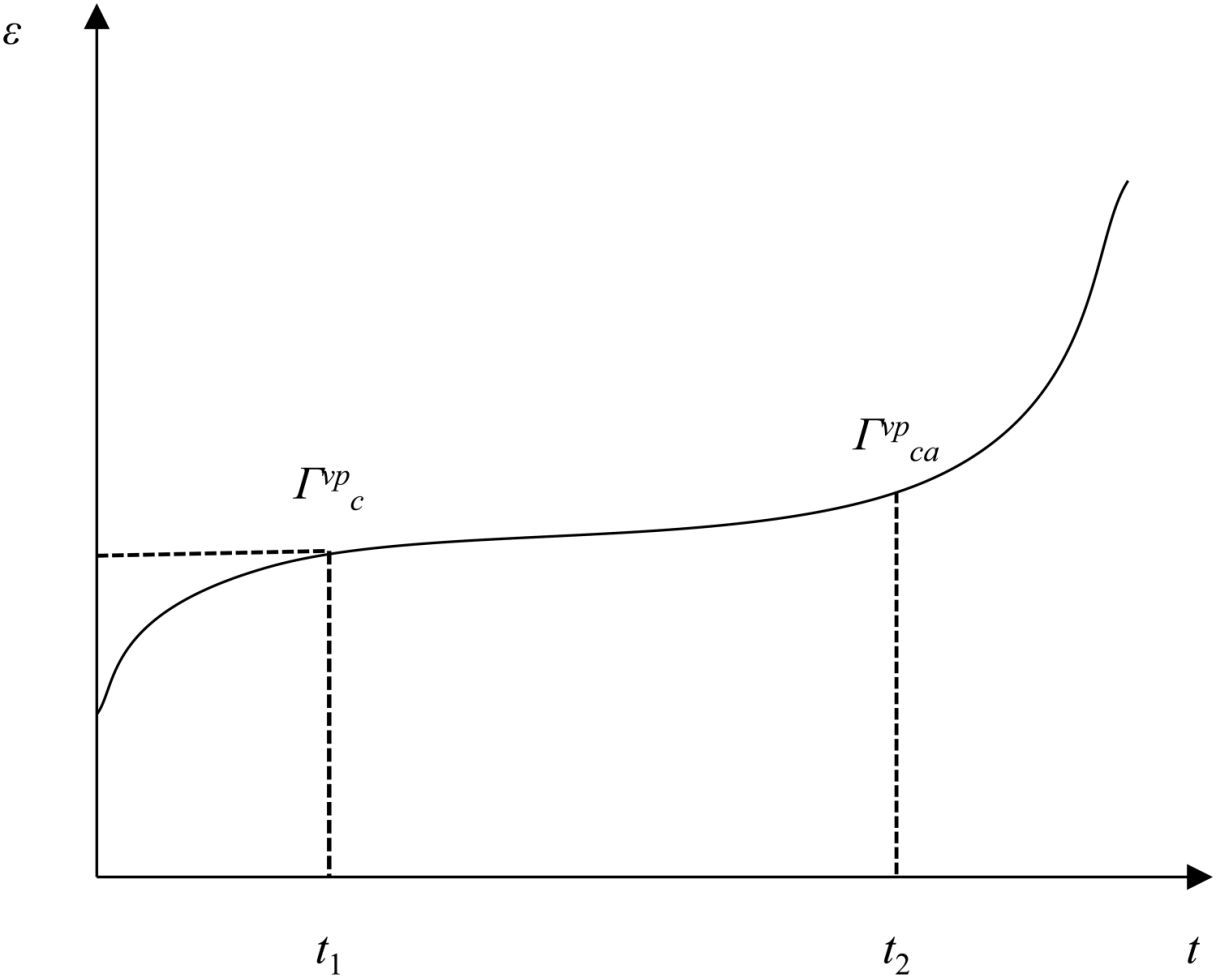
The creep critical strain energy density control threshold [25].

Combined with the characteristics of the whole process of rock creep, the critical strain energy density value is used as the control threshold of different stages in the process of rock creep. Therefore, the critical strain energy density at the critical point of primary creep and steady-stable creep is defined as *Γ*^*vp*^_*c*_. The lower limit is *Γ*^vp*^_c_. Under different stress states, as long as the strain energy density exceeds its critical value, the rock will enter the non-zero constant creep stage. Similarly, the critical strain energy density at the critical point of accelerated creep and steady-stable creep is defined as *Γ*^*vp*^_*ca*_. The lower limit is *Γ*^vp*^_ca_. Under different stress states, as long as the strain energy density exceeds its critical value, the rock will enter the accelerated creep stage. The creep critical strain energy density control threshold is shown in Fig 1 [25].

As shown in Fig 1, the macroscopic creep strain change process of rock is generally divided into three stages. The first section is the primary creep stage. If the creep strain energy density is less than the critical strain energy density of non-zero constant creep, the rock will not enter the constant creep stage. The third stage is that the strain energy density of rock is greater than or equal to the critical strain energy density of accelerated creep, and the rock enters the accelerated creep stage. The second stage is in the steady-stable creep stage between the two. *t*_1_ is the time of the demarcation point between decay creep and steady-stable creep. *T*_2_ is the time of the boundary point between accelerated creep and steady-stable creep. *Γ*^*vp*^_*c*_ is the critical strain energy density at the critical point of primary creep and steady-stable creep. *Γ*^*vp*^_*ca*_ is the critical strain energy density at the critical point of accelerated creep and steady-stable creep.

The arbitrary function of the yield function is defined by the creep strain energy density function [25].


$$\phi \left(F\right)={\left[\frac{\int F\left(\sigma \right)d\varepsilon }{\Gamma_{c}^{vp*}}\right]}^{n}$$ (2)

where *F* is the yield function, *n* is a constant, *σ* is the stress, *ε* is the strain.

The formula for calculating the strain energy density in the linear elastic stage is


$$W=\frac{1}{2}\sigma \varepsilon$$ (3)

where *σ* is the stress,*ε* is the strain,*W* is the strain energy density.

The improved Perzyna viscoplastic model based on the strain energy density is


$$\varepsilon_{vp}^{\prime }=\eta v_{p}\left[\frac{\sigma \varepsilon_{s}}{\sigma_{s}\varepsilon_{sl}}\right]$$ (4)

where *ε*_*s*_ is the creep strain.

The lower limit *Γ*^vp*^_c_ can be written as


$$\Gamma_{c}^{vp*}=\sigma_{s}\varepsilon_{sl1}$$ (5)

where *σ*_*s*_ is the long-term strength, *ε*_*sl*1_ is the strain corresponding to the boundary point of steady-stable creep and primary creep.

The lower limit *Γ*^vp*^_c_ can be written as


$$\Gamma_{ca}^{vp*}=\sigma_{s}\varepsilon_{sl2}$$ (6)

where *ε*_*sl*2_ the strain corresponding to the boundary point of steady-stable creep and accelerated creep.

According to the literature [26,27], when *t*_1_ < *t* < *t*_2_, creep deformation can be expressed as


$$\varepsilon_{s}=a_{1}+b_{1}\ln t+c_{1}t$$ (7a)

where *a*_1_, *b*_1_, and *c*_1_ are test parameters.

When *t* ≥ *t*_2_, the creep deformation can be expressed as


$$\varepsilon_{s}=a_{2}+b_{2}\ln t+c_{2}t++dt^{e}$$ (7b)

where *a*_2_, *b*_2_, *c*_2_, *d* and *e* are test parameters.

Because the test curve has the characteristics of three stages of creep, the initial creep stage, the steady-state creep stage and the accelerated creep stage. The combination of logarithmic function and polynomial function can more flexibly fit the curve change trend of different stages. The logarithmic function has certain advantages in describing the rapid change and asymptotic characteristics of the initial stage. The polynomial function can accurately fit the slope and curvature changes of the curve at different stages by adjusting the coefficient. Therefore,it can better simulate and describe the complex characteristics of sandstone in the whole creep process. It provides a powerful mathematical tool for studying the mechanical behavior and engineering application of sandstone.

When *Γ*^vp*^_c_ < *W* < *Γ*^vp*^_ca_ and *σ* < *σ*_*s*_, the viscoplastic creep deformation can be expressed as


$$\varepsilon_{vp1}^{\prime }=\eta_{vp1}\left[\frac{\sigma \left(a_{1}+b_{1}\ln t+c_{1}t\right)}{\sigma_{s}\varepsilon_{sl1}}\right]$$ (8)

The initial conditions are *t *= 0, *ε*_*vp*_ = 0. By integrating Eq. (8), we get


$$\varepsilon_{vp1}=\frac{\sigma \eta_{vp1}t}{2\sigma_{s}\varepsilon_{sl1}}\left(2a_{1}+c_{1}t+2b_{1}\ln t-2b_{1}\right)$$ (9)

When *W* ≥ *Γ*^vp*^_ca_ and *σ* ≥ *σ*_*s*_, the viscoplastic creep deformation can be expressed as


$$\varepsilon_{vp2}^{\prime }=\eta_{vp2}\left[\frac{\sigma \left(a_{2}+b_{2}\ln t+c_{2}t++dt^{e}\right)}{\sigma_{s}\varepsilon_{sl2}}\right]$$ (10)

The initial conditions are *t *= 0, *ε*_*vp*_ = 0. By integrating Eq. (10), we get


$$\varepsilon_{vp2}=\frac{\sigma \eta_{vp2}t}{2\sigma_{s}\varepsilon_{sl2}}\left(2a_{2}+c_{2}t+2b_{2}\ln t-2b_{2}+\frac{2dt^{e+1}}{e+1}\right)$$ (11)

In general, the instantaneous strain *ε*_*e*_ of rock can be described by Hooke’s law.


$$\varepsilon_{e}=\frac{\sigma }{E_{e}}$$ (12)

where *E*_*e*_ is the instantaneous elastic modulus.

The viscoelastic strain can be expressed as


$$\varepsilon_{ve}=\frac{\sigma }{E_{ve}}\left[1-\exp(-\frac{E_{ve}}{\eta_{ve}}t)\right]$$ (13)

where *E*_*ve*_ is the viscoelastic modulus, and *η*_*ve*_ refers to viscosity coefficients.

The total strain *ε* can be expressed as [28]


$$\varepsilon =\varepsilon_{e}+\varepsilon_{ve}+\varepsilon_{vp}$$ (14)

where *ε*_*e*_ is the instantaneous strain, *ε*_*ve*_ is the viscoelastic strain, and *ε*_*vp*_ is the viscoplastic strain.

By substituting Eqs.(9), (11), (12) and (13) into Eq.(14), we get

When *W* < *Γ*^vp*^_ca_ and *σ* < *σ*_*s*_, the total creep deformation can be expressed as


$$\varepsilon =\frac{\sigma }{E_{e}}+\frac{\sigma }{E_{ve}}\left[1-\exp(-\frac{E_{ve}}{\eta_{ve}}t)\right]$$ (15)

When *Γ*^vp*^_c_ < *W* < *Γ*^vp*^_ca_ and *σ* < *σ*_*s*_, the total creep deformation can be expressed as


$$\varepsilon =\frac{\sigma }{E_{e}}+\frac{\sigma }{E_{ve}}\left[1-\exp(-\frac{E_{ve}}{\eta_{ve}}t)\right]+\frac{\sigma \eta_{vp1}t}{2\sigma_{s}\varepsilon_{sl1}}\left(2a_{1}+c_{1}t+2b_{1}\ln t-2b_{1}\right)$$ (16)

When *W* ≥ *Γ*^vp*^_ca_ and *σ* ≥ *σ*_*s*_, the total creep deformation can be expressed as


$$\begin{array}{c} \varepsilon =\frac{\sigma }{E_{e}}+\frac{\sigma }{E_{ve}}\left[1-\exp(-\frac{E_{ve}}{\eta_{ve}}t)\right]+\frac{\sigma \eta_{vp1}t}{2\sigma_{s}\varepsilon_{sl1}}\left(2a_{1}+c_{1}t+2b_{1}\ln t-2b_{1}\right)+\\ \frac{\sigma \eta_{vp2}t}{2\sigma_{s}\varepsilon_{sl2}}\left(2a_{2}+c_{2}t+2b_{2}\ln t-2b_{2}+\frac{2dt^{e+1}}{e+1}\right) \end{array}$$ (17)

In summary, a new nonlinear creep model of rock is obtained.

### 2.2 Creep model under three-dimensional stress state

Rock is a three-dimensional stress state in the actual engineering environment. In underground engineering such as tunnel excavation and mining, the rock is subjected to stress from different directions. One-dimensional or two-dimensional creep models cannot fully reflect the deformation characteristics of rock under such complex stress environment. For example, in deep underground engineering, rocks are not only subjected to gravity in the vertical direction, but also to in-situ stress in the horizontal direction. The three-dimensional creep model can consider the coupling relationship between these stresses in different directions. More realistic simulation of the actual stress and deformation of the rock.

The stress tensor *σ*_*ij*_ can be decomposed into spherical stress tensor *σ*_*m*_ and deviatoric stress tensor *S*_*ij*_. Similarly, strain tensor *ε*_*ij*_ can be decomposed into spherical strain tensor *ε*_*m*_ and deviatoric strain tensor *e*_*ij*_ [29].


$$\left\{ \;\;\;\begin{array}{c} \sigma_{ij}=S_{ij}+\delta_{ij}\sigma_{m}\\ \varepsilon_{ij}=e_{ij}+\delta_{ij}\varepsilon_{m} \end{array}\right.$$ (18)

where *δ*_*ij*_ is the Kronecker function, the spherical stress tensor *σ*_*m*_=(*σ*_1_ + 2*σ*_3_)/3, the spherical strain tensor *ε*_*m*_=(*ε*_1_ + 2*ε*_3_)/3, and *S*_*ij*_ = *σ*_*ij*_* − σ*_*m*_.


$$\left\{ \;\;\;\begin{array}{c} S_{ij}=2Ge_{ij}\\ \sigma_{m}=3K\varepsilon_{m} \end{array}\right.$$ (19)

where *G* is the initial shear modulus, and *K* is the initial bulk modulus.

According to the analogy method and Eqs. (12), (18) and (19), we get


$$\varepsilon_{ij}^{e}=\frac{1}{2G_{e}}S_{ij}+\frac{1}{3K}\sigma_{m}\delta_{ij}$$ (20)

The axial strain of rock under three-dimensional stress state is


$$\varepsilon_{11}^{e}=\frac{1}{2G_{e}}S_{11}+\frac{1}{3K}\sigma_{m}$$ (21)

The variables *S*_11_ and *σ*_*m*_ can be expressed as


$$\left\{ \;\;\;\begin{array}{c} \sigma_{m}=\frac{\sigma_{1}+2\sigma_{3}}{3}\\ S_{11}=\frac{2\left(\sigma_{1}-\sigma_{3}\right)}{3} \end{array}\right.$$ (22)

By substituting Eq. (22) into Eq. (21), we obtain


$$\varepsilon_{11}^{e}=\frac{\sigma_{1}-\sigma_{3}}{3G_{e}}+\frac{\sigma_{1}+2\sigma_{3}}{9K_{e}}$$ (23)

where is *G*_*e*_ is the shear modulus of instantaneous strain, *K*_*e*_ is the bulk modulus of instantaneous strain, *ε*_11_^*e*^ is the instantaneous strain.

According to the analogy method and Eqs. (13),(18)and (19),we get


$$\varepsilon_{11}^{ve}=\frac{S_{11}}{2G_{ve}}\left[1-\exp\left(-\frac{2G_{ve}}{2H_{ve}}t\right)\right]$$ (24)

By substituting Eq. (22) into Eq. (24), we obtain


$$\varepsilon_{11}^{ve}=\frac{\sigma_{1}-\sigma_{3}}{3G_{ve}}\left[1-\exp\left(-\frac{G_{ve}}{H_{ve}}t\right)\right]$$ (25)

where *ε*_11_^*ve*^ is the viscoelastic strain, *G*_*e*_ is the shear modulus of viscoelastic strain, *H*_*ve*_ is the viscosity coefficient of viscoelastic strain.

However, the viscoplastic strain cannot be directly transformed by analogy. Under normal temperature and medium- and low-temperature conditions, hydrostatic pressureis generally believed to exert a minimal effect on creep, whereas stress deviation plays a major role in creep. Thus, the yield function can take the following form [30].


$$F=\sqrt{J_{2}}-\frac{\sigma_{s}}{\sqrt{3}}=\frac{\sigma_{1}-\sigma_{3}-\sigma_{s}}{\sqrt{3}}$$ (26)

where *J*_2_ is the second invariant of the stress tensor, *σ*_1_ is axial strain and *σ*_3_ is confining pressure.

When *Γ*^vp*^_c_ < *W* < *Γ*^vp*^_ca_ and *σ* < *σ*_*s*_, the viscoplastic creep deformation under the three-dimensional stress state can be expressed as


$$\varepsilon_{11}^{vp1}=H_{vp1}\left[\frac{F\left(a_{1}+b_{1}\ln t+c_{1}t\right)}{\sigma_{s}\varepsilon_{sl1}}\right]=H_{vp1}\frac{\left(\sigma_{1}-\sigma_{3}-\sigma_{s}\right)\left(a_{1}+b_{1}\ln t+c_{1}t\right)}{\sqrt{3}\sigma_{s}\varepsilon_{sl1}}$$ (27)

When *W* ≥ *Γ*^vp*^_ca_ and *σ* ≥ *σ*_*s*_, the viscoplastic creep deformation under the three-dimensional stress state can be expressed as


$$\varepsilon_{11}^{vp2}=H_{vp2}\left[\frac{F\left(a_{2}+b_{2}\ln t+c_{2}t++dt^{e}\right)}{\sigma_{s}\varepsilon_{sl2}}\right]=H_{vp2}\frac{\left(\sigma_{1}-\sigma_{3}-\sigma_{s}\right)\left(a_{2}+b_{2}\ln t+c_{2}t++dt^{e}\right)}{\sqrt{3}\sigma_{s}\varepsilon_{sl2}}$$ (28)

The total strain *ε* under the three-dimensional stress state can be expressed as


$$\varepsilon_{11}=\varepsilon_{11}^{e}+\varepsilon_{11}^{ve}+\varepsilon_{11}^{vp}$$ (29)

where *ε*_*e*_ is the instantaneous strain under the three-dimensional stress state, *ε*_*ve*_ is the viscoelastic strain under the three-dimensional stress state, and *ε*_*vp*_ is the viscoplastic strain under the three-dimensional stress state.

By substituting Eqs. (20), (21), (23) and (24) into Eq. (25), we get

When *W* < *Γ*^vp*^_ca_ and *σ* < *σ*_*s*_, the total creep deformation under the three-dimensional stress state can be expressed as


$$\varepsilon_{11}=\frac{\sigma_{1}-\sigma_{3}}{3G_{e}}+\frac{\sigma_{1}+2\sigma_{3}}{9K_{e}}+\frac{\sigma_{1}-\sigma_{3}}{3G_{ve}}\left[1-\exp\left(-\frac{G_{ve}}{\eta_{ve}}t\right)\right]$$ (30)

When *Γ*^vp*^_c_ < *W* < *Γ*^vp*^_ca_ and *σ* < *σ*_*s*_, the total creep deformation under the three-dimensional stress state can be expressed as


$$\varepsilon_{11}=\frac{\sigma_{1}-\sigma_{3}}{3G_{e}}+\frac{\sigma_{1}+2\sigma_{3}}{9K_{e}}+\frac{\sigma_{1}-\sigma_{3}}{3G_{ve}}\left[1-\exp\left(-\frac{G_{ve}}{\eta_{ve}}t\right)\right]+H_{vp1}\frac{\left(\sigma_{1}-\sigma_{3}-\sigma_{s}\right)\left(a_{1}+b_{1}\ln t+c_{1}t\right)}{\sqrt{3}\sigma_{s}\varepsilon_{sl1}}$$ (31)

When *W* ≥ *Γ*^vp*^_ca_ and *σ* ≥ *σ*_*s*_, the total creep deformation under the three-dimensional stress state can be expressed as


$$\begin{array}{c} \varepsilon_{11}=\frac{\sigma_{1}-\sigma_{3}}{3G_{e}}+\frac{\sigma_{1}+2\sigma_{3}}{9K_{e}}+\mathrm{\backslash vspace}1mmH_{vp1}\frac{\left(\sigma_{1}-\sigma_{3}-\sigma_{s}\right)\left(a_{1}+b_{1}\ln t+c_{1}t\right)}{\sqrt{3}\sigma_{s}\varepsilon_{sl1}}+\\ H_{vp2}\frac{\left(\sigma_{1}-\sigma_{3}-\sigma_{s}\right)\left(a_{2}+b_{2}\ln t+c_{2}t++dt^{e}\right)}{\sqrt{3}\sigma_{s}\varepsilon_{sl2}}+\frac{\sigma_{1}-\sigma_{3}}{3G_{ve}}\left[1-\exp\left(-\frac{G_{ve}}{\eta_{ve}}t\right)\right] \end{array}$$ (32)

In summary, a new nonlinear creep model of rock under the three-dimensional stress state is obtained.

## 3 Indoor creep test

**Fig 2:**
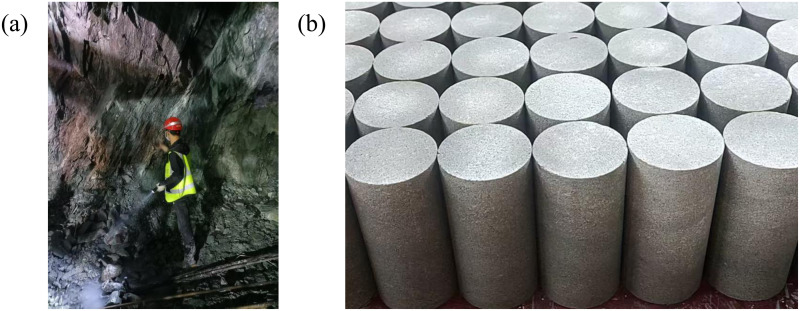
Roadway face and rock samples. (a) Photos of the palm of the scene; (b) rock sample.

The rock is taken from a roadway in Dandong City, Liaoning Province(As shown in Fig 2(a)). The buried depth of the tunnel face is about 600 m. The measured in-situ stress at this location is 9.15 ~ 11.36 MPa. For the convenience of test loading, the confining pressure value is 10 MPa. The sample is prepared into a cylinder with a height of 100 mm and a diameter of 50 mm (As shown in Fig 2(b)). The samples with different water content are obtained by soaking the rock for different time.

The conductivity of the test water is ≤ 0.5mS/ m at 25 °C. This shows that the ion content in the tertiary water is relatively low, which can meet the basic requirements of water quality purity in general chemical analysis experiments, and will not interfere with the experimental results due to too many ions in the water. After evaporation at 105 °C ± 2 °C, the residue content was less than 2 mg/ L. This means that the content of non-volatile substances in water is low, which avoids the interference of these substances in the experiment due to concentration during the experiment. When the pH value is 25 °C, the pH value range is 6.5–7.0. This pH range is close to neutral, which can meet the requirements of most general chemical analysis experiments on the acidity and alkalinity of water.

### 3.1 Indoor triaxial mechanical properties test

The indoor triaxial mechanical properties test steps are as follows [30].

(1) The processed rock samples are placed on the base of the triaxial pressure chamber. It must be ensured that the axis of the specimen coincides with the axis of the axial loading system. The deviation does not exceed ± 0.2 mm. The rubber sealing sleeve is installed around the sample to prevent the leakage of the confining pressure medium. It is necessary to ensure that the rubber sleeve and the sample are closely bonded. And it is necessary to avoid the uneven confining pressure during the test.
(2) The confining pressure medium is slowly injected into the pressure chamber through the confining pressure loading system to uniformly apply the confining pressure. The confining pressure loading rate is generally controlled to 0.01MPa/ s. It is necessary to avoid the dynamic load effect inside the sample caused by excessive loading, which affects the test results.
(3) In the process of applying confining pressure, the change of confining pressure recorded by the data acquisition system is observed. When the confining pressure reaches the set value, the confining pressure is kept stable for a period of time, so that the sample is fully deformed and stable under the action of confining pressure. At the same time, check the sealing of the pressure chamber and whether the data acquisition system is working properly.
(4) After the confining pressure is stable, the axial load is applied to the sample through the axial loading system. The loading method can be controlled by displacement. The loading rate is usually set at 0.001 mm/ s. During the axial loading process, the data changes of axial load, axial displacement and confining pressure are continuously recorded.
(5) It is necessary to observe the residual deformation of the sample under confining pressure. Then it is necessary to slowly release the confining pressure and end the test. At the same time, the axial stress-axial strain curve is drawn in real time through the data acquisition system to intuitively understand the mechanical behavior of the sample during the loading process.

**Fig 3:**
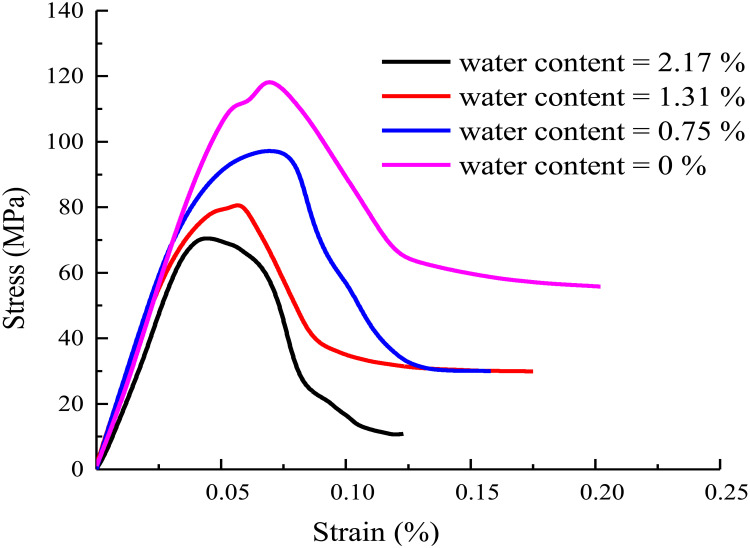
The axial stress-strain curves.

The axial stress-strain curves are shown in Fig 3.

From Fig 3, it can be seen that with the increase of water content, the peak strength of rock decreases continuously. When the rock is in a low water content state, the water content in its internal pores and micro-cracks is less. At this time, the friction between rock particles is relatively large, and the combination between particles is relatively close. In the triaxial compression test, when the rock with low water content is subjected to axial pressure, it is necessary to overcome the large inter-particle friction to produce deformation and failure. Therefore, its peak intensity is relatively high. As the water content increases to a medium level, the pore water content inside the rock increases. Moisture will form a water film on the surface of rock particles, which plays a certain lubrication role and reduces the friction between particles. Under the action of triaxial stress, rock particles are more prone to relative sliding and dislocation, resulting in a decrease in the peak strength of the rock. When the water content of the rock is at a high level, a large amount of water is filled in the pores and fissures of the rock. On the one hand, the presence of water further weakens the connection force between rock particles. On the other hand, the increase of pore water pressure will produce outward thrust on rock particles, which will reduce the effective stress between rock particles. In the triaxial test, the rock with high water content will undergo obvious deformation and failure under low axial pressure, and its peak strength will be greatly reduced. Therefore, the change of water content changes the friction and connection force between rock particles. The water content has a significant effect on the peak strength of rock. In the practice of rock mechanics engineering, it is very important to accurately consider the influence of water content for reasonably evaluating the stability and safety of rock engineering.

### 3.2 Indoor triaxial creep test

In this paper, the single specimen is loaded step by step. The specific triaxial creep test steps are as follows.

(1) The specimen is installed in the pressure chamber of the triaxial testing machine to ensure that the specimen is in close contact with the inner wall of the pressure chamber. The sample is fixed on the pressure chamber loading platform.
(2) The confining pressure needs to be loaded to a predetermined value. The confining pressure loading rate is generally controlled to 0.01MPa/ s. It is necessary to ensure that the confining pressure remains unchanged throughout the loading process.
(3) The axial load is divided into several levels. The initial load is 50% of the peak strength of the rock under the corresponding conditions. The difference between the adjacent two levels of load is 10% of the peak strength. After each stage of loading, the load is kept constant, and the deformation of the sample is continuously observed until the deformation rate is stable, and then the next level of load is applied.
(4) When the deformation of the sample reaches the specified limit, the test ends. Before the end of the test, the axial load and confining pressure need to be gradually reduced to avoid the failure of the sample during the unloading process.
(5) The data recorded during the test are analyzed, and the creep curve is drawn to study the creep characteristics and failure mechanism of the sample.

**Fig 4:**
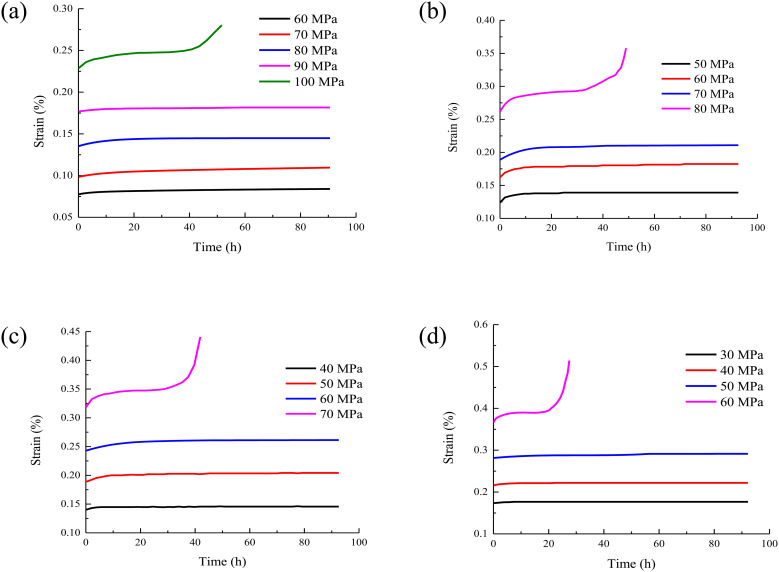
The creep duration curve of rock under different water content. (a) water content 0%; (b) water content 0.75%; (c) water content 1.31%; (d) water content 2.17%.

The creep duration curve of rock under different water contents are drawn as shown in Fig 4.

It can be seen from Fig 4 that the increase of water content will lead to the increase of instantaneous strain of rock at the moment of applying load. This is because the presence of water molecules increases the distance between rock particles, increases the initial porosity of the rock, and is more prone to deformation under load. The steady-state strain rate refers to the rate at which the strain increases steadily with time during creep. The increase of water content will increase the steady-state strain rate of rock. It means that in the stable stage of creep, the deformation speed of rock will also be accelerated by the increase of water content. Higher water content will shorten the creep failure time of rock. Because the presence of water accelerates the damage evolution process inside the rock, the rock reaches the failure state faster under long-term load.

With the increase of water content, the creep deformation of rock is also increasing. This is because the uneven distribution of water in rock will lead to stress concentration. When the water content of the rock increases, the difference in the distribution of water inside the rock may be more obvious. In some areas, the water content is high and the strength decreases significantly. While in other areas, the water content is relatively low and the strength is relatively high. In this way, under the action of load, the stress will concentrate to the areas with low strength, which will aggravate the deformation of these areas. It result in the increase of creep deformation of the whole rock. In addition, the increase of pore water pressure will also change the effective stress state inside the rock. It will change the actual stress of the rock and further affect the creep deformation of the rock.

With the increase of rock moisture content, the creep curve time and accelerated creep time under the last load decrease, which is mainly due to the following reasons. Water has a softening effect on rock, which will reduce the strength and stiffness of rock. When the water content increases, the cementing material between the mineral particles in the rock is softened, and the connection force between the particles is weakened. The overall structure of the rock becomes more loose. Under the action of load, the expansion of micro cracks and the generation of new cracks are more likely to occur in the rock. Therefore, the rock can reach the failure state faster. As a result, the creep curve time and accelerated creep time are shortened. At the same time, water plays a lubricating role in the pores and fissures of the rock. With the increase of water content, the friction between the particles in the rock decreases, and the particles are more prone to relative sliding and displacement. Under the action of load, this lubrication effect makes the deformation of rock easier to develop and accelerates the creep process of rock. It shortens the time to reach the accelerated creep stage and the time of the whole creep curve.

**Fig 5:**
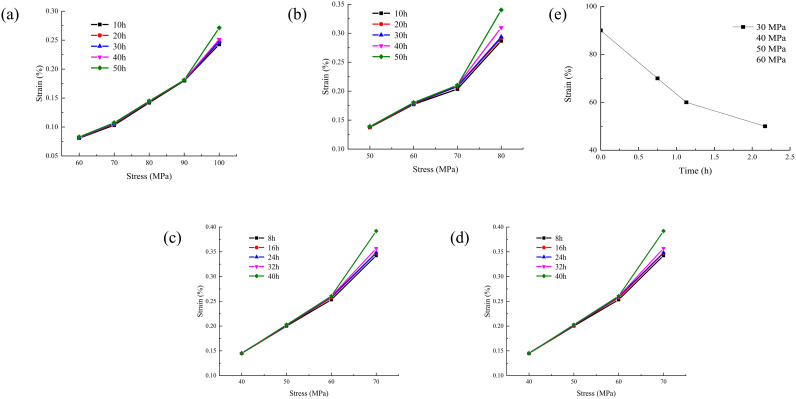
The isochronous stress-strain curve of rock under different water contents. (a) water content 0%; (b) water content 0.75%; (c) water content 1.31%; (d) water content 2.17%.

According to the axial creep duration curve [31], the isochronous stress-strain curve of rock under different water contents are drawn as shown in Fig 5.

It can be seen from Fig 5 that the isochronous stress-strain curve refers to the curve of stress to creep strain in the rectangular coordinate at a specified time after the test load is applied. It can be seen from Fig 4 that the curve is a cluster of divergent lines. The curves almost coincide before the divergence point, and the mechanical properties of the rock show a linear relationship. After the divergence point, the curve begins to diverge, and the rock mechanical properties show a nonlinear relationship. The stress corresponding to the divergence point is the long-term strength value of the rock. With the increase of water content, the long-term strength of rock shows a significant downward trend. This is due to the fact that water enters the pores and fissures of the rock, which will generate pore water pressure. This pressure will offset the effective stress between some rock particles, thereby reducing the long-term strength of the rock. At the same time, the presence of water reduces the friction on the surface of the rock particles and weakens the connection force between the particles. It is like adding lubricant to the rock particles, making the rock more prone to deformation and failure. Some mineral components in the rock will be softened by water. For example, clay minerals will expand and soften after encountering water, which reduces the overall strength of the rock. Even some minerals with high hardness may undergo physical and chemical changes in long-term contact with water. It result in a decrease in their mechanical properties. This in turn affects the long-term strength of the rock. After water enters the pores and cracks of the rock, it will continuously wedge into the tiny cracks and defects of the rock under the action of pressure. With the increase of water content, this wedge effect will make the cracks expand and connect, forming a larger crack network. Thus, the microstructure of the rock is destroyed, the bearing capacity of the rock is reduced. And the long-term strength is reduced.

## 4 Parameter identification and model verification

At present, the commonly used method for solving model parameters is the experimental curve fitting least square method. It can be used to determine the parameters of various rheological models. The difference in accuracy is small and very convenient, and the application range is the widest. The Origin has a nonlinear least squares meshing tool. Therefore, this paper uses Origin software to simulate [31]. The creep mechanical parameters are shown in Table 1.

**Table 1:** The creep mechanical parameters.

Stress (MPa)	30	40	50	60
*G*_*e*_ (GPa)	20.56	17.67	14.78	11.9
*K*_*e*_ (GPa)	26.92	23.49	20.07	16.64
*G*_*ve*_ (GPa)	6.63	6.11	5.59	5.07
*H*_*ve*_ (GPa·h)	5.29	6.63	7.97	9.31
*H*_*vp*1_ (GPa·h)	8.33	9.14	10.21	11.32
*a*_1_	3.58	4.86	6.06	7.24
*b*_1_	1.32	1.84	2.21	2.71
*c*_1_	0.76	1.01	1.26	1.51
*H*_*vp*2_ (GPa·h)	6.17	6.98	8.21	9.49
*a*_2_	6.48	8.69	11.11	13.08
*b*_2_	2.66	3.61	4.43	5.45
*c*_2_	1.13	1.51	1.92	2.28
*d*	16.51	18.08	19.30	21.01
*e*	7.27	7.81	8.58	9.08
*R* ^2^	0.98	0.96	0.97	0.97

**Fig 6:**
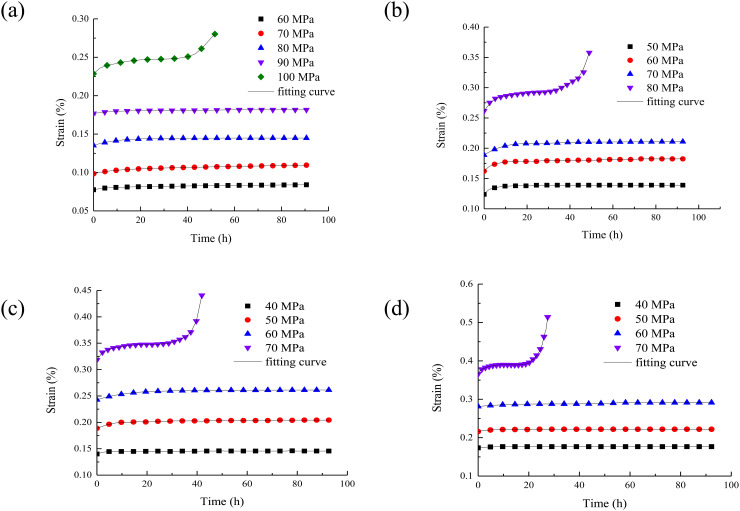
The comparison between the test curve and the model curve of different water contents. (a) water content 0%; (b) water content 0.75%; (c) water content 1.31%; (d) water content 2.17%.

The comparison between the test curve and the model curve of different water contents are shown in Fig 6.

It can be seen from Fig 6 that the overall fitting degree of creep model curve and test curve is better under different water content. The model curves are in good agreement with the experimental curves, especially in the initial and stable stages of creep. The model can reflect the characteristics of the acceleration stage to a certain extent, but there may be some errors in predicting the time point of failure. This is because the rapid expansion and interconnection of internal cracks in rock are affected by many factors, which makes the difference between the model and the test curve more obvious at this stage. The correlation coefficient is an indicator to measure the strength of the linear relationship between the two variables, ranging from-1–1. When the correlation coefficient is close to 1, it shows that there is a strong positive linear correlation between the two variables. The correlation coefficient between the model curve and the test curve is above 0.95, indicating that the model can well fit the test data, and the predicted results of the model are highly consistent with the actual test results. This means that the model can accurately capture the relationship between the variables involved in the experiment and has a strong ability to explain the object or phenomenon studied. In this case, it is reliable to use this model to describe and predict related physical processes or behaviors, which can provide strong support for further research and analysis.

However, although the model can reflect the characteristics of the acceleration stage to a certain extent, there may be some errors in predicting the time point of failure. This is because the rapid expansion and interconnection of cracks in rock are affected by many factors, which makes the difference between the model and the test curve more obvious at this stage. In practice, the development of cracks in rock is not only related to water content and load, but also related to the inhomogeneity of mineral composition and the complexity of internal microstructure. The difference in mineral composition will lead to different mechanical properties of each part of the rock, thus affecting the crack propagation speed. However, the micro-pores, micro-cracks and other defects in the microstructure are difficult to fully reflect the interaction mechanism in the model under the action of stress. In addition, environmental factors such as temperature change and groundwater flow will also affect the propagation of rock cracks. These factors, which are difficult to quantify and accurately simulate, together cause the error of the model in predicting the failure time point, which makes the model curve deviate from the test curve to a certain extent in the acceleration stage.

**Fig 7:**
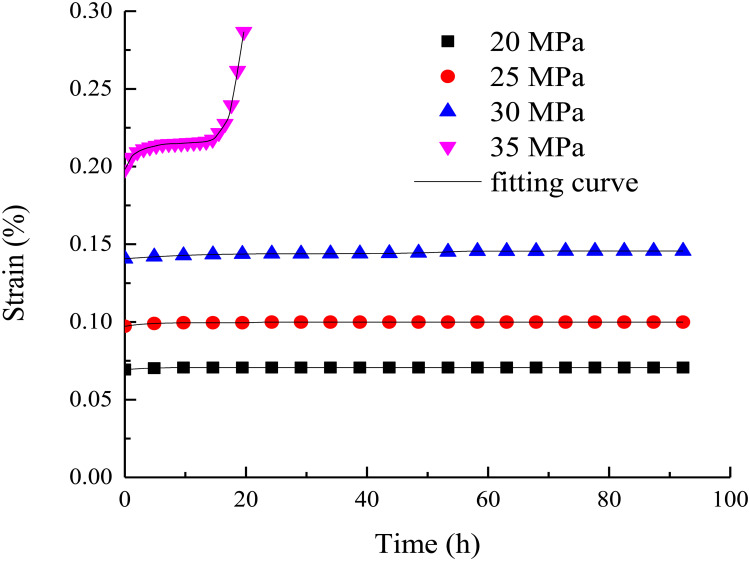
The comparison between the uniaxial creep curves and the model curves.

The rock sample with water content of 0% is the standard sample of rock. However, in order to further prove the applicability of the model, the test data of the rock under uniaxial action are used to verify the model. The comparison between the uniaxial creep curves and the model curves are shown in Fig 7.

It can be seen from Fig 7 that the creep constitutive model curve considering the whole process strain energy density of creep deformation is also in good agreement with the uniaxial creep test curve, and the correlation coefficient is also above 0.90. This further indicates that the creep constitutive model considering the strain energy density of the whole process of creep deformation has high reliability and accuracy. It shows that the model can accurately describe the deformation behavior of rock under uniaxial creep, and can well reflect the actual creep process. This provides strong support for the analysis and prediction of rock creep characteristics in rock mechanics related research and engineering, and increases its credibility and practicability in practical applications.

**Fig 8:**
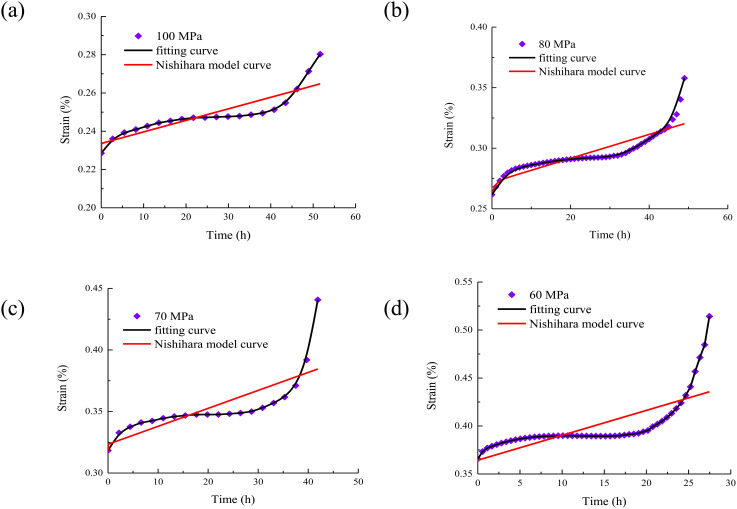
The creep duration curve of rock under different water content. (a) water content 0%; (b) water content 0.75%; (c) water content 1.31%; (d) water content 2.17%.

In order to study the superiority of the established model, the goodness of fit between the Nishihara model and the existing model is compared. The comparison of the Nishihara model curve, the existing model curve and the test curve is shown in Fig 8.

It can be seen from Fig 8 that the coincidence between the creep constitutive model curve and the test curve considering the whole process of creep deformation is better than that between the Nishihara model curve and the test curve. The model can better capture the actual mechanical behavior of the material in the creep process and more accurately reflect the creep deformation characteristics of the material. It shows that the description of the internal micro-mechanism of the material is more in line with the actual situation, and can more accurately reflect the strain response of the material under different stress and time conditions. Due to the better agreement with the experimental curve, it means that the model has higher accuracy in predicting the creep deformation and strain energy density change of the material. For practical engineering applications, more reliable prediction can provide stronger support for structural design, life assessment, etc., and reduce the risks and errors caused by inaccurate models.

**Fig 9:**
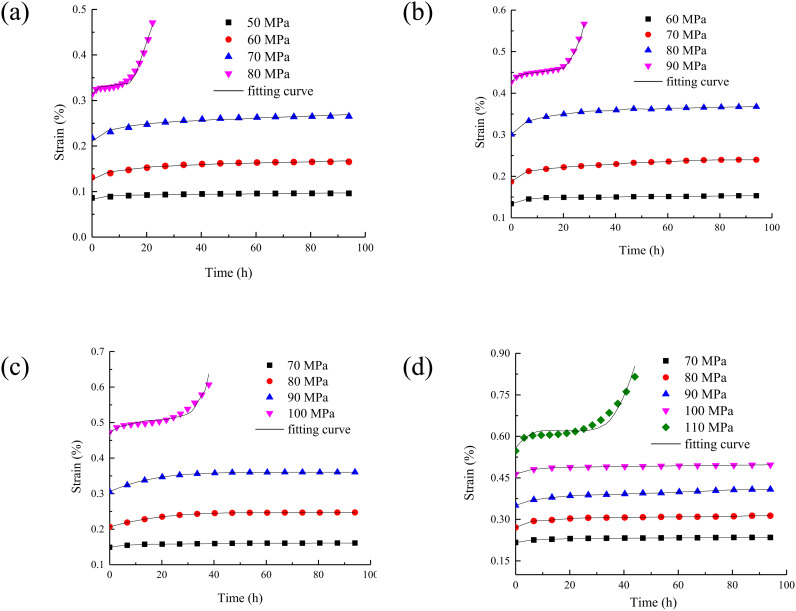
The test curve and model curve of sandstone in the literature [32]. (a) confining pressure 10 MPa; (b) confining pressure 13 MPa; (c) confining pressure 16 MPa; (d) confining pressure 19 MPa.

In order to verify that the model established in this paper is suitable for other types of rocks, the model is verified by the experimental data of sandstone in the literature [32]. The test curve and model curve of sandstone in the literature [32] are shown in Fig 9.

It can be seen from Fig 9 that the creep constitutive model considering the whole process of creep deformation is not only applicable to the creep curve of rock samples in this paper. It also well describes the creep deformation law of sandstone in the literature. It shows that the model has universal applicability to different types of rock materials and can be used to study and analyze the creep behavior of various rock materials. It can perform well in different rock materials, indicating that the model is not occasionally applicable to a specific rock, but can grasp some common characteristics and internal laws of rock creep deformation. Its theoretical basis and mathematical expression are scientific and reasonable. It can accurately reflect the mechanical behavior of rock materials in the creep process, and provide reliable theoretical support for related research and engineering applications in the field of rock mechanics.

## 5 Sensitivity analysis of model parameters

**Fig 10:**
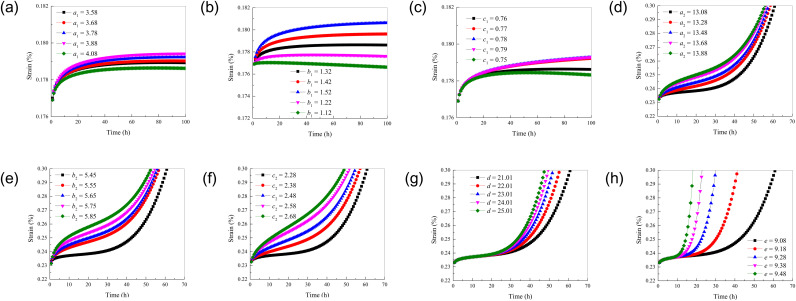
The creep deformation laws of rock under different parameters. (a) parameter *a*_1_; (b) parameter *b*_1_; (c) parameter *c*_1_; (d) parameter *a*_2_; (e) parameter *b*_2_; (f) parameter *c*_2_; (g) parameter *d*; (h) parameter ***e.***

The influence of different parameters on the creep characteristics of rock is introduced by parameter sensitivity analysis. The creep deformation laws of rock under different parameters are shown in Fig 10.

From Fig 10, it can be seen that with the continuous increase of parameter *a*_1_, the decay creep rate and the final deformation of stable creep are increasing. With the increase of the parameter *b*_1_, the decay creep rate, the stable creep rate and the final deformation of the stable creep increase. As the parameter *c*_1_ increases, the decay creep rate and the stable creep rate increase. With the increase of parameter *a*_2_, the steady creep rate increases. As the parameter *b*_2_ increases, the decay creep rate and the stable creep rate increase. As the parameter *c*_2_ increases, the decay creep rate and the stable creep rate increase. With the increase of parameter *d*, the accelerated creep rate increases and the creep time decreases. With the increase of parameter *e*, the accelerated creep rate increases and the creep time decreases. At this time, the creep curve of the acceleration stage is almost vertical.

It shows that *a*_1_ can promote the cumulative deformation of rock in the stable creep stage, which may be that it changes the plastic deformation ability of rock under long-term stress. Therefore, the rock can produce greater final stable deformation. The parameter *b*_1_ affects the mechanism of dislocation movement or grain boundary sliding in the stable creep stage of rock. The deformation rate of rock in the stable stage is accelerated. It may be that *a*_2_ has a specific effect on the micro-deformation mechanism inside the rock during the stable creep stage. In the attenuation creep stage, *b*_2_ may be involved in the initial adjustment process of the internal microstructure of the rock. It affects the friction between particles or the fracture and recombination of chemical bonds. Thus, the deformation rate is accelerated. The mechanism of *c*_2_ may be similar to that of *c*_1_. In the decay creep stage, *c*_2_ may affect the energy dissipation or stress concentration during the initial adjustment of the internal microstructure of the rock. It result in an accelerated deformation rate. It is shown that *d* and *e* have a significant effect on the deformation behavior of the rock entering the accelerated creep stage. They may affect the accumulation rate and expansion mechanism of micro-damage inside the rock, making the rock reach the failure state in a shorter time. The larger *d* and *e* may accelerate the initiation, propagation and coalescence of cracks in the rock. They result in a sharp increase in the deformation rate and a shortening of the creep time, thus making the creep curve in the acceleration stage steeper.

## 6 Conclusions

The established three-dimensional creep constitutive model was verified by using the creep test data of rock samples with different axial pressures and different water contents. According to the creep parameters obtained by inversion, it is found that the established creep constitutive model can completely simulate the whole stage of rock creep, especially the acceleration stage of rock creep. It makes up for the defects of the traditional creep model, and also verifies the accuracy and rationality of the established model.

Based on the strain energy theory of continuous medium, the critical strain energy density value is used to describe the characteristic quantity of sudden change of creep mechanical behavior of materials. The accelerated creep time determined by this method can not only reflect the influence of cumulative creep strain, but also effectively reflect the influence of stress state on accelerated creep, which provides the possibility of predicting accelerated creep, and the number of tests required to determine the parameters of constitutive model is greatly reduced, which provides convenience for practical application.

With the increase of water content, the long-term strength of rock shows a significant downward trend. This is due to the fact that water enters the pores and fissures of the rock, which will generate pore water pressure. This pressure will offset the effective stress between some rock particles, thereby reducing the long-term strength of the rock. At the same time, the presence of water reduces the friction on the surface of the rock particles and weakens the connection force between the particles. It is like adding lubricant to the rock particles, making the rock more prone to deformation and failure.
